# Altered kidney function in fatty liver disease: confronting the “MAFLD-renal syndrome”

**DOI:** 10.3389/fcdhc.2024.1539117

**Published:** 2025-01-08

**Authors:** Suleiman Al Ashi, Ali A. Rizvi, Manfredi Rizzo

**Affiliations:** ^1^ Endocrinology Fellow, UCF COM HCA Healthcare GME – Endocrinology, Diabetes, and Metabolism Fellowship Orlando VA Healthcare System, Orlando, FL, United States; ^2^ Department of Medicine, Division of Endocrinology, Orlando VA Medical Center and University of Central Florida College of Medicine, Orlando, FL, United States; ^3^ School of Medicine, Promise Department, University of Palermo, Palermo, Italy; ^4^ Department of Medicine, Ras Al Khaimah (RAK) Medical and Health Sciences University, Ras Al Khaimah, United Arab Emirates

**Keywords:** metabolic dysfunction-associated fatty liver disease (MAFLD), renal dysfunction, metabolic syndrome, type 2 diabetes, obesity

**Figure 1:**
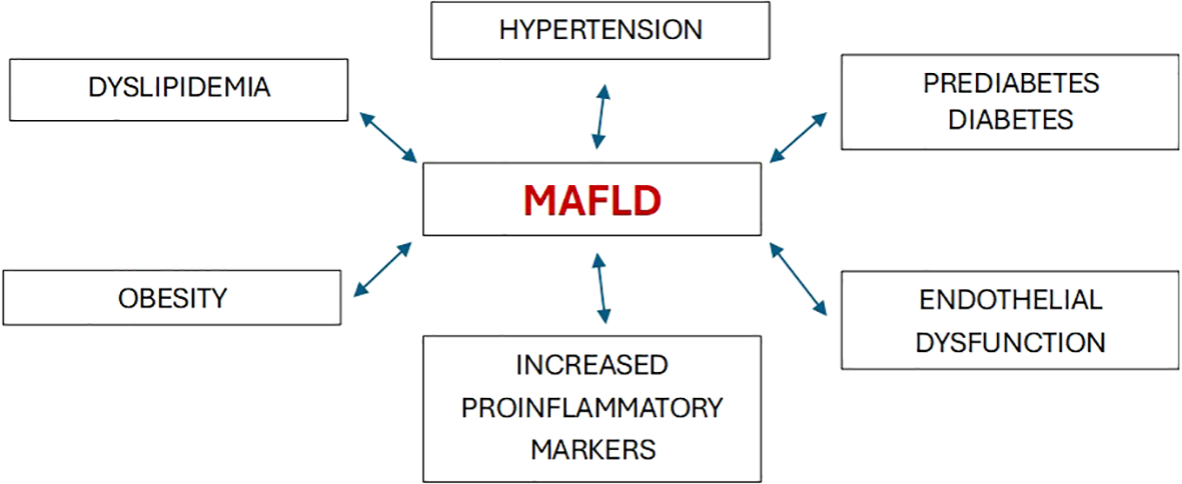
Relationships between MAFLD and metabolic disorders that increase cardiovascular risk.

Nonalcoholic fatty liver disease (NAFLD) is one of the most common causes of chronic liver disease globally. In 2020, a new terminology, namely “metabolic dysfunction-associated fatty liver disease” (MAFLD), was proposed (1). Cardiometabolic criteria have been added in the updated definition to highlight the elevated cardiovascular risk in these patients. The revised definition better emphasizes the central role of metabolic dysfunction in the development and progression of this highly prevalent condition. From a morbidity standpoint, both definitions are associated with an increased risk of developing diabetes, cardiovascular disease, and renal dysfunction. In a study of 6873 individuals with a 4.6-year follow-up, the associations of MAFLD and NAFLD with diabetes, chronic kidney disease (CKD), and cardiovascular disease (CVD) were similar (2). Epidemiological evidence indicates that MAFLD is not only associated with an increased risk of liver-related complications, but also increases the possibility of developing several extra-hepatic diseases, including new-onset type 2 diabetes (T2DM) as well as adverse cardiovascular and renal outcomes (3). Metabolic disorders, including overweight/obesity, T2DM, hypertension, and dyslipidemia are often associated with systemic organ dysfunction, thereby suggesting that similar morbidity could occur in MAFLD (**Figure 1**). The novel understanding underscores the bidirectional relationship between hepatic steatosis and the metabolic dysfunction continuum.

The association between MAFLD and renal dysfunction is intriguing and has been highlighted in recent years. MAFLD has been proposed to be independently associated with an increased risk of CKD (4). Parvanova and colleagues investigated the prevalence of MAFLD in prediabetes, visceral obesity, and preserved kidney function, and explored whether MAFLD is associated with renal hyperfiltration, an early sign of kidney damage (5).

Renal hyperfiltration is generally defined as an estimated glomerular filtration rate (eGFR) that is 2 standard deviations above the age- and sex-specific mean, i.e.: ≥98th percentile. They analyzed data from more than 6000 Spanish civil servants, aged 18-65 years, with fasting plasma glucose ≥ 100 and ≤ 125 mg/dL (prediabetes). Approximately 4000 patients (62.9%) had MAFLD, and 330 (4.9%) had renal hyperfiltration. MAFLD was more frequent in individuals with hyperfiltration than without (86.4% vs 61.7%, P < 0.001). More than half of the subjects with prediabetes, visceral obesity, and estimated glomerular filtration rate (eGFR) ≥ 60 ml/min presented with MAFLD that was associated with hyperfiltration.

Interestingly, the pathophysiologic basis of the association between MAFLD and altered renal function has still to be fully unraveled. A recent finding is that MAFLD is associated with systemic dysregulation (6). It is regarded by some as the hepatic manifestation of the metabolic syndrome (MetS), with heightened inflammation, altered renal hemodynamics, elevated proinflammatory markers, and endothelial dysfunction all playing a part (7). In the setting of increased oxidative stress and a chronically abnormal adipokine profile, the low-grade, subclinical inflammation promotes hepatic lipid accumulation and atherogenic dyslipidemia (“lipotoxicity”). In this regard, the roles of two key adipocytokines in MAFLD are worth mentioning. Increased leptin secretion promotes progression of steatosis to steatohepatitis, with a concomitant reduction in adiponectin, which has anti-inflammatory anti-atherogenic properties (8). Increased visceral fat deposition also enhances release of tumor necrosis factor-a (TNF-a) and interleukin-6 (IL-6) (9). The aforementioned factors have been shown to contribute to an increased likelihood of kidney involvement in individuals with MAFLD (10).

MAFLD and glomerular hyperfiltration share common risk factors, including obesity, insulin resistance (IR), impaired glucose tolerance, dyslipidemia, and hypertension. Abbate et al. found an increased association between MAFLD and dysglycemia (11). They reported that the prevalence of MAFLD averaged 19.3%, and progressively increased from 14.7% to 33.2% and 48.9% in subjects with normoglycemia, prediabetes and T2DM, respectively. Chen et al. aimed to clarify the association between MAFLD and incident end-stage renal disease (ESRD) prospectively in a cohort of 337,783 UK Biobank participants over a median duration of 12.8 years (12). Participants with MAFLD were twice likely to develop ESRD, and the association of MAFLD with ESRD risk remained significant in both non-CKD and CKD participants. Since hepatic IR is a common accompaniment of MAFLD, it is not surprising that T2DM shows a significant epidemiologic overlap with the latter. Worldwide, between 18%-33% of individuals with NAFLD also have T2DM, while up to 66%-83% of those with fatty liver disease have varying degrees of IR (13, 14). Clinical improvements in MAFLD and T2DM can favorably impact CKD progression (15).

Studies have shown that the coexistence of MAFLD and CKD predicts the risk of ischemic heart disease (IHD) better than MAFLD or CKD alone (16). In addition, the combination of abdominal obesity and MAFLD increases the prevalence of, and mortality from, CKD. In a retrospective cohort study of 9161 participants that analyzed the National Health and Nutrition Examination Surveys III (NHANES III) data from 1988 to 1994, the abdominally obese MAFLD group had the highest all-cause mortality as well as disease-specific mortality during the 30-year follow-up period (17). Abdominal obesity could therefore serve as a mediator in the association between MAFLD and CKD.

Ascertaining the relationship between CKD and the development of liver fibrosis in MAFLD is of obvious and significant clinical importance. In this respect, individuals with CKD exhibited a greater incidence of fibrosis compared to those without CKD (75.6% vs. 24.4%) (18). Liver fibrosis as assessed by transient elastography is independently associated with albuminuria in MAFLD subjects (19). It is noteworthy that liver steatosis was found to be a better predictor of CKD than fibrosis in MAFLD (20).

Having explored the association between MAFLD and CKD, we would like to recommend early and more aggressive measures to favorably impact the natural history of the “MAFLD-Renal Syndrome”. The main clinical interventions are listed in **Table 1**. Firstly, the primary importance of early identification of MAFLD, while still in the reversible stages, in a population with increasing rates of obesity and type 2 diabetes cannot be over-emphasized. This requires a high index of suspicion and involves screening asymptomatic high-risk patients for this condition using approved tools. The diagnosis of MAFLD or the presence of its associated metabolic risk factors should be promptly followed by evaluation for kidney dysfunction. Such an early screening strategy is justified based on the evidence showing an epidemiologic, and likely a pathophysiologic, association between the two conditions. It would be reasonable to employ sensitive measurements of early renal damage, such as microalbuminuria, estimated glomerular filtration rate (eGFR) and emerging biomarkers such as nystatin C. Lifestyle interventions including dietary modifications, weight loss, and physical activity are beneficial for amelioration of the obesity-metabolic syndrome and form the therapeutic cornerstone of management (21). Pharmacologic interventions aimed at weight loss and MAFLD are likely to be helpful to the kidneys, although a detailed review is beyond the scope of this paper. There is preliminary evidence supporting the use of nutraceutical approaches and certain MiRNAs in mitigating the deleterious milieu and modifying gene expression in steatohepatitis (22, 23). The thyroid hormone receptor activator *resmetirom* is the only drug approved to reduce liver fat accumulation and treat noncirrhotic hepatitis and moderate-to-advanced hepatic fibrosis in MAFLD (24); however, its impact on the progression of associated renal dysfunction is unknown. It is evident that preventive aspects of early intervention are key to reducing the burden of renal disease in MAFLD. In this context emerging areas of research and therapeutics include the following: 1) the potential role of the renin-angiotensin system in the genesis and progression of renal dysfunction in MAFLD (25); 2) the role of immune mechanisms such as antigen-activated CD8+ T cells, and the impact of immune-modulating agents, in the pathogenesis of MAFLD-associated renal disease (26), and 3) the applicability of several promising renal biomarkers, such as kidney injury molecule-1 (KIM-1) and liver-type fatty acid binding protein (L-FABP), in the detection and surveillance of renal involvement in fatty liver disease (27). Future efforts also need to be directed at investigating and targeting the unique hepatorenal pathways that operate in this evolving pathophysiologic relationship.

**Table 1:** Recommended renoprotective measures in MAFLD.

Screening and Testing
Serum Creatinine
Estimated Glomerular Filtration Rate
Urine Microalbumin and Protein Excretion
Imaging: Ultrasound and/or Computed Tomography

Therapeutic Interventions
Lifestyle Changes: Mediterranean-type Diet, Weight Optimization, Physical Activity
Attaining and Maintaining Glycemic, Blood Pressure, and Lipid Targets
Use of Proven Pharmacologic Agents
